# Personalised, predictive and preventive medication process in hospitals—still rather missing: professional opinion survey on medication safety in Czech hospitals (based on professional opinions of recognised Czech health care experts)

**DOI:** 10.1186/1878-5085-5-7

**Published:** 2014-05-01

**Authors:** Zeno Veselik

**Affiliations:** 1 ABC Works CZ s. r. o., U Kříže 632/24, Praha 5 - Jinonice, 158 00 Prague, Czech Republic

**Keywords:** Predictive, Preventive, Personalised Medication, safety, ADE, CPOE, Unit-dose Packaging, ADMS

## Abstract

The survey had the following aims: (1) to rationalise the hypothesis that risks and losses relating to medication process' errors in Czech hospitals are at least comparable with the other developed countries and EU countries especially, (2) to get a valid professional opinion/estimate on the rate of adverse drug events happening in Czech hospitals, (3) to point out that medication errors represent real and serious risks and (4) to induce the hospital management readiness to execute fundamental changes and improvements to medication processes. We read through a lot of studies inquiring into hospitals' medication safety. Then, we selected the studies which brought reliable findings and formulated credible conclusions. Finally, we addressed reputable Czech experts in health care and asked them structured questions whether the studies' findings and conclusions corresponded with our respondents' own experience in the Czech hospital clinical practice and what their own estimates of adverse drug events' consequences were like. Based on the reputable Czech health care expert opinions/estimates, the rate of a false drug administration may exceed 5%, and over 7% of those cause serious health complications to Czech hospital inpatients. Measured by an average length of stay (ALOS), the Czech inpatients, harmed by a false drug administration, stay in hospital for more than 2.6 days longer than necessary. Any positive changes to a currently used, traditional, ways of drug dispensing and administration, along with computerisation, automation, electronic traceability, validation, or verification, must well pay off. Referring to the above results, it seems to be wise to follow the EU priorities in health and health care improvements. Thus, a right usage of the financial means provided by the EC—in terms of its new health programmes for the period 2014–2020 (e.g. Horizon 2020)—has a good chance of a good result in doing the right things right, at the right time and in the right way. All citizens of the EU may benefit using the best practice.

## Review

### Introduction

It is surprising that—despite tremendous progress in pharmaceutics, diagnostics, surgery or other medical technologies—there has been hardly any advance in medication process in Czech (European) hospitals for many decades, as though the technology and progress avoided the medication processes. The reality is, however, contrary to fact that medication has a much wider choice in drugs with much stronger treatment effects than those a couple of years ago. However, progress in medicament efficiency also brings much stronger, undesirable, or excessive side effects at the same time. This is one of the reasons why a medication of inpatients in hospitals may be considered quite a risky process which has been documented and proven by a number of serious studies performed worldwide. Most of the damage is caused by human factor failures, i.e. errors made during a traditional drug administration process used in prevailing part of EU hospitals and currently in all hospitals of the Czech Republic. In order to transfer the international experience into the Czech health care environment and to show that this subject is important for both the inpatients' safety and their treatment efficiency, I have initiated and performed a special survey based on the Czech Republic reputable experts' opinions.

### Topic-related overview

At the end of 2012, the ‘General report & recommendations in predictive, preventive and personalised medicine 2012: white paper of the European Association for Predictive, Preventive and Personalised Medicine’ [1] was issued. It was a kind of a good international momentum initiating a new approach to modern medicine perception, definition and institutionalisation. The White Paper also comprises the ‘Patient-Specific Modelling’ part, which depicts the multifunctional and interdisciplinary approach necessary for clinical application of personalised medicine. The individualised patient records and patient models, individualised therapy with medical/technical systems and IT-assisted processes represented the major topics, closely related to this article contents.

Errors and adverse events happen in all kinds and forms of medical care, or all types of health care workplaces. That is the reason why it is not surprising that there is a wide spectrum of studies using different methodologies and bringing a wide range of results. All the studies performed have one point in common—the errors and adverse events are a very frequent and considerable problem in all types of institutions, where the errors were tracked [2] (Table 1).

**Table 1:** Some examples of extensive studies' results (focused on adverse event incidence in providing health care)

**Country of the study realisation**	**Year(s) of realisation**	**Number of patients involved**	**Number of adverse events recorded**	**Percentage of the adverse events**
USA (HMPS)	1984	30,195	1,133	3.8
USA (UCMPS)	1992	14,565	475	2.9
Australia	1992	14,179	2,353	16.6
Great Britain	1999–2000	1,014	119	11.0
Denmark	1998	1,097	176	9.0
New Zealand	1998	6,579	849	12.9
Canada	2001	3,720	279	7.5
Brazil	2003	1,103	84	7.6
Sweden	2003–2004	1,967	241	12.3

The European Commission (EC) pays quite a lot of attention to the patient safety and quality of health care. There are good reasons for this. Based on its own studies performed in recent years, EC found out that an estimated 8%–12% of patients admitted to hospitals in the EU suffer from adverse events whilst receiving health care (e.g. health care-associated infections amounting to approximately 25% of adverse events, *medication-related errors*, surgical errors, medical device failures, errors in diagnosis and failure to act on the results of tests). Much of the patients' harm is preventable, but the implementation of strategies to reduce harm varies widely across the EU [3]. The present article focuses on the medication-related errors and their consequences, and it suggests some possibilities to prevent them efficiently.

Other extensive studies confirm that failures and errors happening during therapy represent quite a serious problem with serious consequences. The ‘Harvard Medical Practice Study’ [4] reviewed 30,121 randomly selected case records of inpatients hospitalised during 1984 in 51 randomly chosen hospitals providing acute health care (excluding psychiatric departments) in the state of New York. Adverse events were defined as damage caused by the therapy and treatment used in providing health care, leading to a patient damage and/or an extension of the patient's hospitalisation. Such adverse events were found at 3.7% of all hospitalisation cases, and 27.6% of them were caused by inadvertency of the care-providing personnel. The adverse drug events were the most frequently occurring non-surgical errors; the diagnostics' failures, errors in therapy care, etc. followed. Of all identified adverse events, 6.5% lead to patients' permanent disability, and 13.6% to patients' death. Two years later, the same team performed a follow-up study focused on preventing medical injury and found that 69.6% of the adverse events mentioned were preventable.

The Utah and Colorado medical practice study [5] done in 1992 assessed 14,052 randomly selected closed-case records in randomly chosen hospitals (Utah and Colorado, USA). The study proved that 2.9% of the inpatients had been harmed by adverse events, while about 30% had been caused by neglect, and about 50% had been preventable. The surgical errors represented 44.9%, the adverse drug events totalled to 19.3%. Of all the adverse events, 8.4% lead to a patient's permanent disability, and 6.6% of the harmed patients died.

Formerly elaborated (in 1974), The California Medical Insurance Feasibility Study [6] similarly identified, in 20,864 finished case records, 4.65% patients who suffered a preventable health damage by their medical therapy. In 1992, ‘The quality in Australian healthcare study’ [7] found that adverse events happen in 16.6% of all hospitalisations, while 51% of them were marked as preventable. The majority of errors were found in surgical procedures, 13.6% in diagnostics, 12.0% in therapy and 10.8% in adverse drug events. In consequence of adverse events, 13.7% of inpatients were permanently disabled, and 4.9% of the inpatients died.

In order to find a way how to draw attention to improve medication safety, we focused on the adverse drug event incidence. The adverse events caused by medication errors that harm inpatients during their hospitalisation represent a specific group of preventable failures. Researching and analysing various studies on medication safety, medication errors, or adverse drug events [8-25], we have tried to spot such studies which provided well-footed results identifying the following:

1. rate of the adverse drug event (ADE) incidence in hospitals using a traditional process of medication (none or not fully used, computerised patient order entry (CPOE), a decentralised non-automated drug administration, none or rare electronic bedside verifications of correct dispensing),

2. rate of ADE causing a serious health damage to hospital inpatients,

3. consequences of serious health damage caused by ADE (in terms of an extended stay of a patient in hospital and/or the extended care induced by ADE).

Generally, all the studies we read through had one feature in common. The more consistent and thorough the studies' observations, the higher rate of adverse drug events and the more serious consequences of them were registered. Seeking the ADE in a form of a well-executed study, we have identified three. Each of them provided the required ADE information and answered one of the above mentioned questions.

### Reference study 1: performed by the Municipal Hospital in Munich in 2004

**Figure 1:**
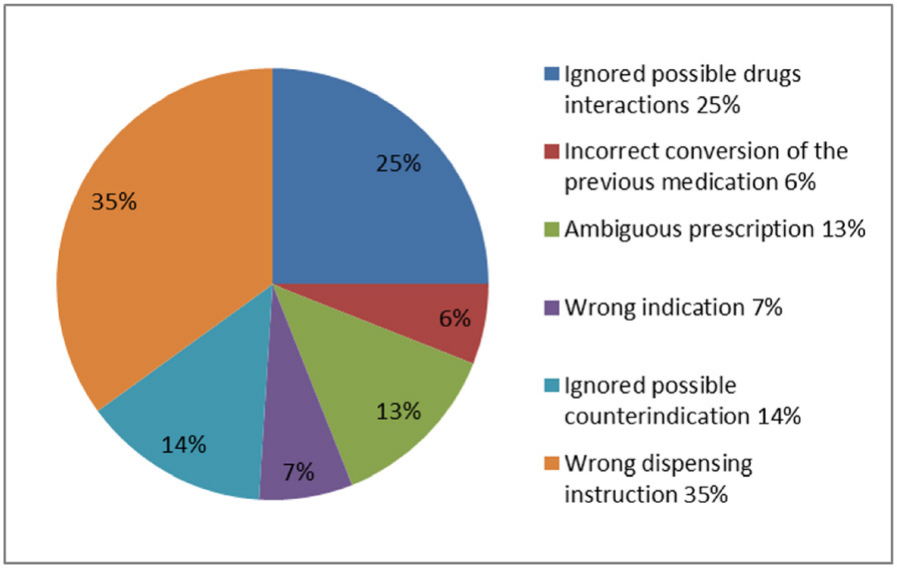
Types and percentage share of errors recorded.

The study was performed by the Municipal Hospital in Munich (Städtisches Krankenhaus München-Harlaching) in 2004 [26]. More information about the study can be found at http://www.adka.de/solva_docs/341_altersberger_poster_weimar.pdf. It focused on medication error incidence especially. It was an inspection or rather examination performed by the hospital for themselves in order to detect their own errors in medication and to identify possibilities for improvements. It was performed for 1 day at 12 wards. The prescriptions and administration of 868 items of drugs to 189 patients (4.6 drug items per patient) were examined. The study focused on both the errors in drug prescription (first part) and the errors in drug administration (second part). Out of the 868 drug item prescriptions, 76 had some errors (8.8%) as shown in Figure 1.

The types of wrong dispensing instructions were further detailed in Table 2.

**Table 2:** Wrong dispensing instructions in detail

**Instruction**	**Percentage**
Wrong dosage	10
Wrong interval for dispensing	10
Wrong medication tenure	10
No reflection of the patient's liver and kidney testing	5

**Figure 2:**
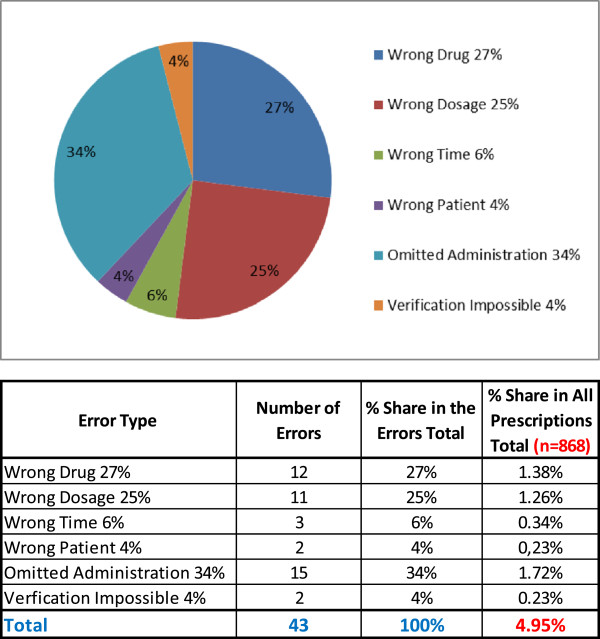
Errors made during drug dispensing.

The errors made during the drug dispensing were assessed in the second part of the study. The hospital pharmacist visited the relevant hospital departments without any prior notice and checked and recorded divergences from the given prescription. The assessment was performed directly, and/or after the drug administration to a patient, and it brought the results shown in Figure 2 and Table 3.

**Table 3:** Errors committed during drug dispensing

**Error type**	**Number of errors**	**Percentage share of the total of errors**	**Percentage share of the total prescriptions (*n*** **= 868)**
Wrong drug	12	27	1.38
Wrong dosage	11	25	1.26
Wrong time	3	6	0.34
Wrong patient	2	4	0.23
Omitted administration	15	34	1.72
Verification was impossible	2	4	0.23
Total	43	100	4.95

*Of all the drugs prescribed, 4.95*% *were wrongly administered*, and at least one of the above defined errors was made, i.e. there was a breach of rules and patients' rights set by the Joint Commission International (JCI). We have also selected and used this study as reference study 1 because the German hospitals (as well as the Czech ones) widely use a traditional, decentralised and manual drug dispensing at their hospital wards. So, the study results may be fairly considered very similarly to those of the Czech hospitals. This study was cited, and its results were used in the ‘Hospital management of the future: knowledge and recommendations of experts’ [27], and there is a good example of a rather shocking impact of medication errors on patients' safety in chapter 26.1 [28]. We used a similar example in the ‘Summary’ section of this article.

### Reference study 2: performed in 36 US hospitals in 2002

Here, we refer to the study ‘Medication errors observed in 36 health care facilities’ performed in 36 USA hospitals in 2002. The results were published in *The Journal of the American Medical Association*[29]. The goal of the study was to identify the prevalence of medication errors (doses administered differently from those ordered). The study was performed as a prospective cohort study on the data from hospitals accredited by the Joint Commission on Accreditation of Healthcare Organizations, non-accredited hospitals, and skilled nursing facilities in Georgia and Colorado. The study participants were selected in a format of a stratified random sample of 36 institutions. Twenty-six declined, with random replacement.

Medication doses given (or omitted) during at least one medication pass during a 1- to 4-day period by nurses on high-medication-volume nursing units. The target sample was 50 day-shift doses per nursing unit or until all doses for that medication pass were administered. Medication errors were witnessed by observation and verified by a research pharmacist (E.A.F.).

Clinical significance was judged by an expert panel of physicians. The study focused on measuring medication errors committed on patients, and it came to the following results: In 36 institutions, *19*% *of the doses (605/3,216) were erroneous*. The most frequent errors by category were wrong time (43%), omission (30%), wrong dose (17%) and unauthorised drug (4%). Seven percent of the errors were considered as potential adverse drug events. There was no significant difference between error rates in the three settings (*P* = 0.82) or by size (*P* = 0.39). The error rates were higher in Colorado than in Georgia (*P* = 0.04). The main conclusions of the study were following: Medication errors were common (nearly one in every five doses in a typical hospital and skilled nursing facility). Potential clinical significance for each error category in the study results was assessed, as shown in Table 4.

**Table 4:** Clinical significance for each error category

**Error category**	**Significant**		**Total**	**Percent significance within each error category**
	**No**	**Yes**		
Dose	8	1	9	11
Omission	271	17	288	6
Unauthorized drug	24	4	28	14
Wrong dose	84	15	99	15
Wrong form	12	6	18	33
Wrong route	4	1	5	20
Wrong technique	0	1	1	100
Wrong time	224	3	227	1
*Total*	*627*	*48*	*675*	*7*

*The percentage of errors rated potentially harmful was 7*%, or more than 40 errors per day in a typical 300-patient facility. The problem of defective medication administration systems, although varied, was clearly declared as widespread.

### Reference study 3: performed in three hospitals in Germany in 2012

In this case, we refer to the study ‘Costs of Adverse Drug Events in German Hospitals—A Microcosting Study’ [30] by Dominik Rottenkolber, MBR, Joerg Hasford, MD, and Jürgen Stausberg, MD in 2012. The aim of this study was twofold: first, to calculate the direct treatment costs associated with ADEs leading to hospitalisation and second, to derive the excess costs and extra hospital days attributable to ADEs of inpatient treatments in selected German hospitals. While the hospital cost structure, proportions and height in Germany differ from those in Czech Republic, we were ‘only’ interested in the part of the study where it tried to identify the extra days of hospitalisation of patients hurt by adverse drug events.

In 2012, an extensive research as one of the first administrative data-based analyses calculating the economic consequences of ADEs in Germany was performed. The study focused on costs of wrong medication causing adverse drug events and their therapeutic remedies/corrections. It was a retrospective and medical record-based study performed from the hospitals' perspective based on administrative accounting data from three hospitals in Germany (49,462 patients were hospitalised there in 2008). Analysis used clinical, demographic and economic data in order to describe the patient sample and calculate their therapy costs. Classification of diagnoses referred to the German modification of ICD-10 used for coding inpatients of German hospitals. The average length of stay (ALOS) of the patients was between 6.8 to 8.7 days which is similar to the ALOS typical of patients of Czech hospitals. One of the study outcomes important for our survey purposes was that the *average length of extra hospitalisation stay caused by adverse drug events was 2.9 days* (Table 5).

**Table 5:** Length of extra hospitalisation stay caused by adverse drug events

**Length of stay**	**Cases (*n*** **= 1,891)**	**Control subjects (*n*** **= 1,891)**	**Difference**
Mean ± SD	12.7 ± 17.2	9.8 ± 11.6	2.9
Median (Q1–Q3)	8.0 (4.0–15.0)	7.0 (3.0–13.0)	1.0
Range	0–273	0–161	–

The study results highlighted considerable extra costs resulting from ADEs. So, the authors of this study, as well as the authors of the other studies mentioned in this article, believe that these study results may support health-policy decision makers in allocating research grants assessing the impact of ADEs on European health care systems, to help find efficient provisions predicting or preventing ADEs or pushing ahead personalised and safe medication procedures for the EU citizens/patients.

When thinking about mitigation, and/or elimination of the above risks, you will come to a couple of common denominators, like computerised physician's order entry, structured medication, data electronic digitalisation, bar coding, two-dimensional coding and automation. Many serious studies give us quite a clear message.

For instance, the study ‘Effect of bar-code technology on the safety of medication administration’ [31] proved that the implementation of structured medication and bar coding in drug management had

•completely eliminated errors arising during transcriptions of physicians' order entries,

•decreased errors in a timely drug administration by 27%,

•lowered all the other types of errors in drug administration by 41%,

•brought down the potential incidence of ADEs by 51%.

However, even a highly computerised medication process cannot bring a total elimination of errors. High rates of ADEs may continue to occur after the implementation of CPOE and the related computerised medication systems that lack decision support for drug selection, dosing and monitoring [32]. On the other hand, the whole portfolio of errors has changed substantially: majority of the errors made during drug administration and drug dispensing has been reduced to a very minimum (administration to 13%, dispensing to 1%, transcription to 0%), while the prevailing part of the remaining errors consists of ordering (61%) and monitoring (25%). Errors in ordering are also preventable, but by means of a pharmacologists' and pharmacists' validation which is effectively contingent on the CPOE and by a possible use of sophisticated software tools. So, the personalisation and electronic digitalisation of data is a must for really modern innovation in medication process and opens the door to an effective interdisciplinary collaboration within major health care processes.

## Methods

Upon discussions about medication safety in hospitals held with a number of reputable Czech health care experts, it was agreed that innovation of a traditionally used medication process in Czech hospitals made good sense, and the topic deserved a special attention. However, as soon as you tried to quantify possible benefits of such innovation, you will find a lack, and/or absence of studies performed in the Czech Republic health care system in a scope and way which would provide reliable and reliable results or conclusions.

As noted in the previous section, there are many studies on the topic of medication errors and their consequences in hospitals performed worldwide, but for the Czech hospital managers and decision makers, the studies represented just something which may be valid in other health care systems and in a different environment, however, not as much in the Czech health care system and its specific environment. The managers have been quite well aware of the time, efforts, workload and financial demands of any change to a traditional, more or less well functioning, medication process used for many decades. When you want to convince the managers of such an investment's pay-off, you needed to come up with something which is reliable and persuasive. In other words, you have to bring good arguments from the Czech hospitals directly, not vicariously.

With no other possibility or sufficient financial funding for elaboration on a Czech thorough study of Czech hospitals, the present author has decided to use high-quality foreign study results and compare them with the Czech hospitals' real situation by means of expert opinions. In order to perform such a comparison, we have addressed reputable Czech experts in health care. We selected those with long-time clinical experience won at various positions held in Czech hospitals (medical doctors/physicians, clinical researchers and scientists, nurses in chief, pharmacists, hospital directors and other distinguished persons, including ministers of health, medical faculty deans, and/or university principal/chancellor).

All the foreign studies selected had the following major characteristics:

•were focused on medication processes identical with or very similar to those used in Czech hospitals,

•used a comprehensive and reliable methodology and accurate and reliable data,

•provided lucid and credible results/conclusions at a rather conservative level.

From a number of studies read through, we have selected those ones which have the above characteristics and provide answers to three crucial questions:

1. What is the rate of medication errors (ADEs) made in hospitals when providing hospital care to inpatients?

2. What is the rate of ADEs which may cause serious negative effects to inpatients' health status, inducing additional extra care or hospitalisation?

3. What are the measurable consequences of ADEs causing serious negative effects to inpatients' health status?

After the above types of study selection, the abstracts have been prepared as well as references to full versions, a structured questionnaire has been created and professional respondents have been asked to answer the following questions:

1. Is the study relevant to a real situation in Czech hospitals?

2. Is the study methodology used for the identification of ADEs reliable and logically correct?

3. Do you believe that the findings, results or conclusions of the study apply to the situation in Czech hospitals?

4. What number or percentage of interval do you consider relevant to and expectable in the Czech hospitals' environment?

### Results

#### Expert opinions on the rate of medication errors

Additional file 1: Table S1 shows an overview of all respondents' answers regardless of their professional status. While the Munich Study came to the 4.9% share of the ADEs out of all medication cases, the Czech experts indicated their opinions based on their experience that in Czech hospitals, the ADE may range from 4.37% (or 3.51%) to 8.65% (5.61%) with a mid-value of 6.51% (5.61%). The numbers in parentheses represent values after exclusion of one piece of the highest and one piece of the lowest values out of all the values obtained. A vast majority of the respondents considered the above reference study with high quality and fully applicable to the Czech environment. The respondents' estimate even showed their opinions that the rate of ADEs in Czech hospitals might be higher than that suggested by the reference study.

Each of respondents had experience, at various positions, in hospital process managements; however, she or he still represented primarily one of four possible professional groups. The four groups were physicians, nurses, pharmacists and managers with non-medical educational backgrounds. Professionally determined views have own quite interesting features. For instance, the insight of experienced nurses present at each procedure of the medication process in person is non-neglectable [Additional file 1]. Without attaching a higher or lower importance to professional group opinions, all interesting and/or complementary information has been shown in the present article.

Physicians apparently saw the above study as relevant to Czech hospitals' real situation, and their estimates of medication error rate reached the mid-values, the same mid-values as those of the above reference study [see Additional file 1: Table S2].

Nurses considered the methodology, realisation and results of the above study fully compatible with their experience in Czech hospitals [see Additional file 1: Table S3]. Their professional estimates of the medication error rate came to much higher values than the above study. Provided you take into account the fact that nurses ensure in person the medication process in hospitals and are familiar with its peculiarities, the estimate has a non-neglectable information value.

Pharmacists considered the above study methodology and quality of its realisation reliable, and its findings relevant for Czech health care's real situation. Their estimates were—in comparison to the study findings—a bit more conservative [see Additional file 1: Table S4).

Also, non-medical managers considered the study methodology and quality of its realisation reliable, and its findings relevant to the Czech health care [see Additional file 1: Table S5). Their estimates were approximately at the level showed by the above study.

## Conclusions

### Conclusion 1

In general, it is obvious that an absolute majority of our respondents consider the Munich study well done, reliable, and with applicable results for practice in Czech hospitals. An important part of the respondents considered a possible rate of medication errors in the Czech environment rather higher than *5*% *out of all the administered drug volume.*

### Expert opinions on the rate of serious impacts caused by wrong medication

As shown in Additional file 1: Table S6, a prevailing majority of respondents considered the above study methodologically correct, well elaborated on, reliable and applicable to the Czech environment (even though performed in a very different health care system). The respondents' estimate of the share of adverse drug events out of the whole hospital medication practice seemed to be slightly higher than that in the above study. The professional groups' views differed a little from each other, and this gives interesting complementary information:

Physicians considered the above study well done, reliable and relevant to the Czech health care environment. Their estimates were rather conservative in comparison to the study findings [Additional file 1: Table S7].

Nurses saw the above study relevant, well done and reliable. Again, their estimates of the adverse drug events' share in medication errors were rather more open and reached almost twofold values in comparison to the other professional groups [Additional file 1: Table S8].

Pharmacists also considered the methodology, quality of realisation, and relevance of the above study very good. Their estimates were rather conservative [Additional file 1: Table S9], similar to the physicians' views.

Non-medical managers viewed the above study's methodology and execution as very good, and its findings as reliable and relevant to the Czech health care environment. Their estimates of the adverse drug events' share in false medications rather concurred with the findings of the study [Additional file 1: Table S10].

### Conclusion 2

Nearly all respondents considered the above study methodologically correct, well and reliably executed, and its results relevant to the Czech health care. Their estimates of the adverse drug events' occurrence in Czech hospitals were around, or slightly above, 7% out of all wrong medication cases committed.

### Expert opinions on the economic impacts of medication errors

A prevailing majority of respondents considered the above study methodologically correct, well executed and relevant to Czech hospitals. The professional estimates of an average length of extra hospitalisation due to damage caused to patients via adverse drug events were slightly more conservative in comparison to the said study, and their average values amounted to approximately 2.6 days [Additional file 1: Table S11].

In terms of the professional groups' view, we saw the following opinions:

Physicians saw the above study methodologically correct, well performed and relevant to the Czech hospitals. Their opinions were a bit more conservative than those of the said study, and they reached an average level slightly above 2.4 days [Additional file 1: Table S12]. Nurses' view was only slightly above the physicians' view, but almost at the same level as that of the study [Additional file 1: Table S13]. Pharmacists' view was very similar to the other professional groups and nearly identical with the above study [Additional file 1: Table S14]. Non-medical managers' view on the quality and methodology used and its relevance was apparently positive. Their opinions on extra hospitalisation length were the lowest ones out of the professional groups surveyed, but it still remained above 2 days of extra hospitalisation [Additional file 1: Table S15].

### Conclusion 3

In general, all respondents agreed on the good quality, good execution and relevance of the above study to Czech hospitals. They also agreed on the probability of extra hospitalisation length exceeding 2 days and on average reaching over 2.6 days.

### Summary

The summary of findings of this survey is as follows:

1. The rate of medication error occurrence in Czech hospitals may be rather high. Based on the respondents' opinion, it will very probably *exceed 5%*! However, in a vast majority, the medication errors have neither been recorded nor reported and, thus, not analysed systematically.

2. Approximately at 5.01%–9.20% (or 5.00%–9.06% after exclusion of extreme values), i.e. on average *at more than 7%, wrongly medicated patients may suffer from serious health complications* in Czech hospitals. Consequently, a corrective therapy requires prolongation of patient's hospitalisation, which produces extra costs to hospitals.

3. *Wrong medication, which will probably cause patients' serious health complications* and induce a corrective therapy, *may be expected to prolong patients' hospitalisation by 2.6 days* or more on average.

The above three findings may be interpreted by means of the following three examples:

1. In a hospital with 1,200 beds, which are occupied by 75%, hospitalisation care is provided to 900 patients. Let us assume conservatively that each of the patients receives seven pieces of a drug a day. That means, they administer 6,300 pieces of a drug a day (note that our hitherto performed analyses at major Czech hospitals have shown that a daily average of the drug pieces administered to patients highly exceeds the value of seven). At least 5% or more of the drug batches administered fail meeting, at minimum, one of six JCI principles for a correct and safe drug administration. That means, more than 315 wrongly administered drug batches/pieces occur a day. In other words, a minimum of 45 patients, or rather up to 315 of the hospital patients, may be hit compromised by a wrong medication case every day.

2. Out of 45–315 patients who may be hit by a wrong medication case a day, 7% may suffer from serious health complications caused by a wrong medication. This represents 3 to 22 patients a day!

3. Considering the above hospital and supposing that the average length of stay, for example, is 8 days, every occupied bed will serve approximately 45 patients a year. That means that the hospital provides hospitalisation to approximately 40,500 patients. Three to 22 patients are hit by a wrong medication a day or approximately 1,100 to 8,000 patients experience serious health complications by wrong medication a year. If a corrective therapy of each of the patients damaged requires 2.6 days of additional hospitalisation, the hospital faces—except for the reputational and legal risks—also economic risks in terms of additional costs. The number of additional hospitalisation days amounts from approximately 2,860 up to 20,800 a year. If we multiply these numbers by costs of one hospitalisation day, we may come from ten to several tens of millions CZK (or EUR million) of extra costs a year. This clearly represents a value which, along with patients' unsafety, is worth of any hospital management's attention.

Based on this survey results, there should not be any doubt that the medication errors represent an important issue for Czech hospitals' managements (as well as for hospital managements all around the world). In terms of the challenges calling for personalisation of medicine, prevention of risks, increasing patients' safety, improving therapy effectiveness, lowering costs of the health care provided, electronic digitalisation of health care data and innovation in health care processes, it is obvious that elimination of errors in hospital medication is a great topic to be addressed and supported by EU funds both at national and—especially—Europe-wide levels.

### Expert recommendations

There are still many processes in health care which have a great potential for improvements and have not been used so far. For this article, we have selected the topic of medication (un)safety in European hospitals with special attention to medication unsafety in hospitals of the Czech Republic. The topic is closely interconnected with patients' treatment safety, therapy effectiveness, drug management versus polypragmasia and drug abuse, integration of pharmacists into the hospital health care process, health care costs, computerised physician order entry, electronic evidence of medication, electronic tracing drugs administration and consumption per patient, lowering work complexity and administrative burden of nurses.

This article is not about inpatients' medication therapy. It is, rather, about a necessary therapy of the medication processes broadly used by our public health care providers (hospitals). The article is also about a need of prevention of errors committed and thus harming our inpatients. And last, but not least, it is about the price we must pay for medication errors in terms of therapy ineffectiveness, useless additional costs of the care and reputation we (may) lose because of the health care unsafety, unless we improve it soon.

It is surprising that—despite tremendous progress in pharmaceutics, diagnostics, surgery or other medical technologies—there has been hardly any advance in medication process in Czech (European) hospitals for many decades. As though the technology and progress avoided the medication processes. The reality is, however, contrary to fact that medication has a much wider choice in drugs with much stronger treatment effects than those a couple of years ago. However, progress in medicament efficiency also brings much stronger, undesirable or excessive side effects at the same time. Unfortunately, contemporary medication is incomparably more costly than the one only 10 years ago.

Nevertheless, prescription as well as administration of drugs is one of the most important processes in providing care in hospitals. Although these processes are rather simple, they are considered the most risky ones in hospitalisation. The reason is a traditional, for many years unchanged, process that provides too many opportunities for occurrence of a number of errors caused by both system imperfections and human factor failures. According to the European Association of Hospital Pharmacists (EAHP), the errors occur over the whole drug administration process, while 39% of errors are made during prescription, 12% during internal information transfer, other 11% during internal logistics and 38% during drug administration [33].

A traditional way of drug dispensing and administration starts with a physician's prescription of a drug to a particular patient. Even though such a prescription is personalised and might be made out electronically, almost 100% of electronic prescriptions made out in Czech hospitals are in a non-structured text format, as observed by the author of this article in his long-term experience in Czech and Slovak hospitals (recently verified and repeatedly re-confirmed). It means that these electronic prescriptions cannot serve as computerised physician's order entry since they lack database data record features which are necessary for further technical elaboration in terms of computerised drug management. No systematic check or supervision over the prescription correctness is done. No systematic attention is paid to patient's current physio-biochemical test results in terms of undesirable interactions and/or suitability of a drug, its form and dosage.

At the moment, the above described prescription (physician's order entry) process is finished; the attending physician completely loses control over any further drug administration or dispensing to a particular patient. The prescription of drugs to patients of a hospital department or ward is collected, usually summarised by a ward nurse in chief, and then—as an aggregate drug order—sent in a paper format or in an electronic text format to the hospital pharmacy.

The hospital pharmacy—based on an aggregate order—prepares a bulk drug shipment. The university graduate pharmacists take over a role of an ordinary stock keeper without any other touch on the real medication process. When the drug shipment to a hospital medical department or ward crosses the hospital pharmacy stock release counter, the hospital loses any central control over the drug stock or drug consumption. When a hospital employee takes over the drug shipment at its pharmacy stock release counter and brings it to a ward, from then on, all drug administration is only in the hands and on the shoulders of nurses. They safe keep the drug stock on wards, prepare individualised batches of drugs to their patients according to physician's prescription and dispense the drugs to the inpatients.

There is no central control over the drug stock, no control over correctness of drug administration, no way to trace both stock and consumption of drugs. Rather, a sad truth in the 21st century. The blame is not on the nurses, of course, but on the system we use. At least 20% or more of all working hours of nurses are spent on preparing drug individual batches (consisting of mechanically pressing out pills from blisters and preparing other drug pieces into drug cups), and other 10% or more of their working time is spent on paperworks necessary for drug administration plus the time of dispensing drugs to patients.

The process is naturally full of mistakes, showing failures in at least one, or rather more, of the JCI principles of correct medication (failing in correct verification of the right patient, right drug, right form, right time, right way/dosage or right documentation).

### Expert recommendation no. 1

Based on the above findings, it seems to be the high time to change a traditional way of administering medication used in the new as well as the other EU member countries' hospitals into a new type to promote personalised medication, prevent medication errors, protect patients' safety, save health care costs, and contribute to high, EU-wide standard of health care safety and effectiveness.

The new type of hospital medication process—in comparison to the traditional one—looks quite different. Most drugs fabricated in bulk may be robotically repacked into unit-dose bags and print-labelled by means of bar coding, or rather two-dimensional types of coding. The prints on the bags with unit-dosed drug comprise all information necessary about the relevant drug in the bag (e.g. drug name, name of the producer, active component(s) denomination, dosage, LOT/batch number and expiration date). A major part of the hospital pharmacy drug and medical material stock is stored in a ‘warehouse module’ of the automated system in a form of variously sized small bags with unit-dosed pieces of drug.

**Figure 3:**
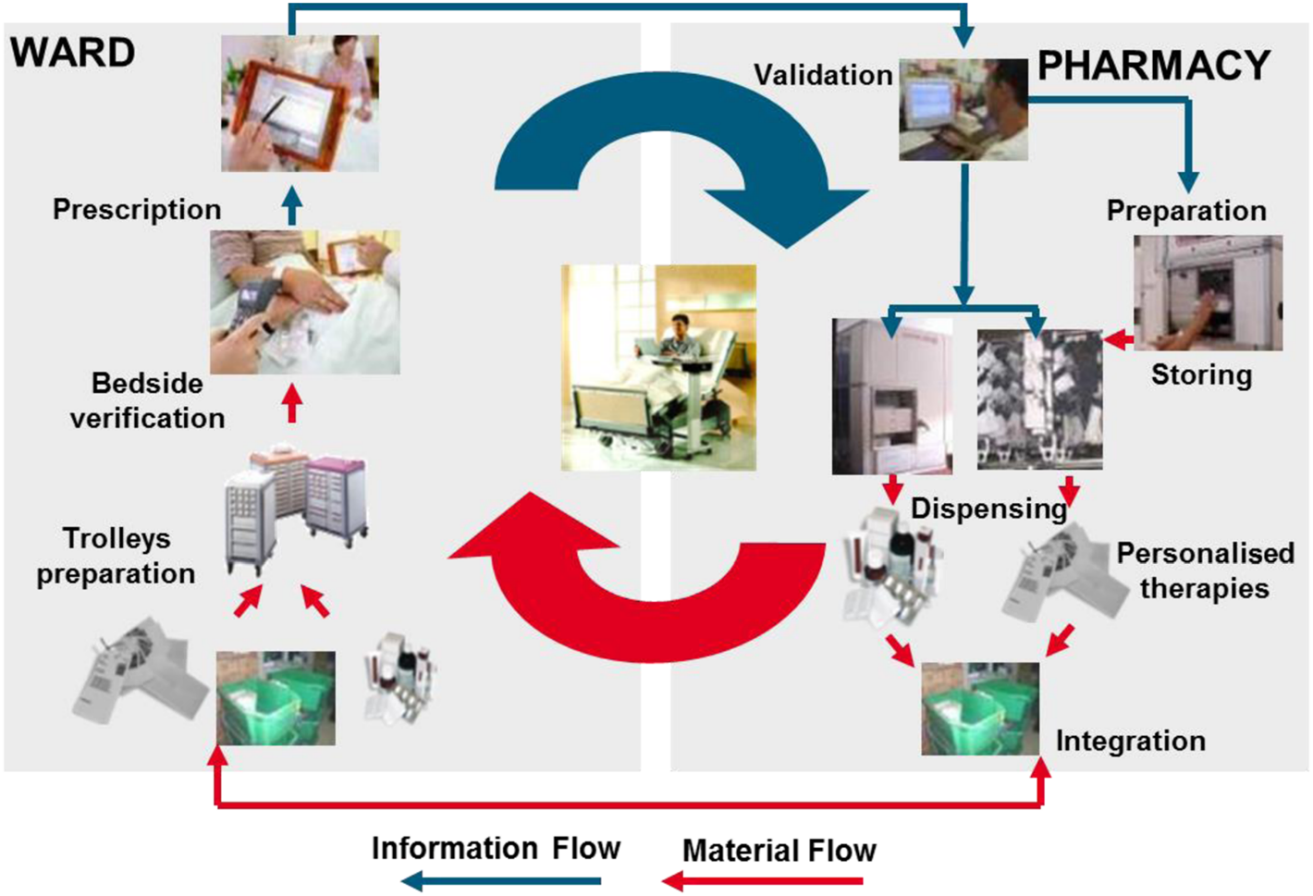
Flowchart of the drug administration process.

A physician prescribes therapy by means of a CPOE; a clinical pharmacist and/or clinical pharmacologist validates the therapy order and launches automated patient specific therapy production. Therapy is automatically produced and delivered to the ward; a nurse verifies and dispenses therapy by means of electronic bedside verification of a patient, drug type, form, dosage and time of application, and thus, she completes right electronic documentation of drug administration. Is it just a utopian vision only? Not really. Let us have a look at Figure 3.

The EAHP claims that just by means of bar coding drugs at unit-dose packaging level, it is possible to eliminate over 41% of errors arising from drug administration [34,35]. Current experience shows that a modern, innovative process as above can remove a vast majority of all the errors occurring in the traditional way of drug administration or dispensing, and it contributes considerably to an elimination of errors in drug administration. The other good news is that the positive effects of such a compact, centralised, automated and fully computerised drug management system change the traditional one substantially in the following aspects:

•the computerised personal electronic order allows for personalised medicine and tracing treatment cost per patient,

•improves the hospital patients' safety and both the medical treatment process's quality and effectiveness,

•eliminates excess costs of patients' longer hospitalisation caused by medication errors,

•decreases wards' drug stock, lowers the overall hospital drug consumption as well as the relevant costs, and provides the hospital management control over important cost items,

•helps the hospital in easier winning and keeping hospitals' accreditations and certifications,

•protects hospitals from potential legal actions taken by patients whose health status was damaged by wrong medication.

The problem is that changing of a long-lasting and rigidly embedded process is very demanding on efforts, courage and confidence of a hospital management. And, it is time consuming and quite costly. In addition, many of the above benefits of the innovative medication system are intangible and/or not quite well visible at first glance (safety, quality, personalised medicine, risks and protection against potential legal actions).

The other problem is that pioneering personalised, preventive, safety-supporting and costs-saving system might not be very well welcome for some reasons and especially in the countries in which there is no good ‘home-grown’ example of anything that is really functioning and bringing indisputably positive results. For a hospital management, it might be risky to start a change which must convert the way of thinking of many colleagues first, which will cost a lot of time and money, will increase traceability and transparency of each patient therapy and cost, will add more responsibility and involvement in medication process on pharmacists and will free about 20% of nurses' working time by means of eliminating their participation in preparation of individualised drug batches to patients.

### Expert recommendation no. 2

Let us use the opportunity of EU international cooperation for personalised, preventive and predictive medicine implementation in terms of experience sharing, know-how transfer and innovative technology implementation support. Such collaboration will show the already-implemented innovative systems and their benefits achieved in the most advanced countries. It also gives the opportunity of experience sharing and best-practice transfer. Data and experience exchange as well as comparing new and traditional models internationally brings more trust and endeavour to those who would like to start with an implementation.

Personally, I have been engaged in an international consulting business in public health care for more than 12 years. Three years ago, I was feeling a lack of new impulses in addressing my clients with new possibilities for improvements to hospital management and health care service provision effectiveness. That was a momentum which brought my consulting firm, ABC Works CZ s.r.o. (Prague, Czech Republic), to cooperate with my colleagues in another consulting firm HC Logic s.r.o. (experts in logistics; Ostrava, Czech Republic). We started to pursue various issues around safety, effectiveness and economy of medication processes in Czech hospitals and approached various Czech hospitals' managements with our findings and recommendations. After several studies we made for Czech hospitals, we may say that we have got quite interesting findings. For instance, there were considerable differences in medication used by Czech hospitals compared, for instance, with the hospitals mentioned in the reference studies used for our opinion survey. Polypragmasia in the Czech health care system seems to be more than an important issue. While the reference hospitals medicated their inpatients by 7–8 units of drugs a day, the Czech hospitals' medication administration was more than twofold. This probably makes our health care more costly as well as represents more risks to inpatients. Also, the spectrum of drugs used in medication of Czech hospitals was quite more extensive (reaching 3,000–4,000 various items in comparison to similar hospitals in France or Germany using 2,000–2,500 items, for instance). The international collaboration and comparing the systems used may bring new impulses into a further development, new inspiration, benchmarks and best practise dissemination.

When considering a change to the traditional way of drug administration in Czech hospitals, the decision makers have always raised reasonable questions. Do the medication error rates suggested by the studies performed in other countries and different health care systems also apply to the Czech hospitals' practice? Is this really a serious problem? Should we invest into a change of our traditional medication process? Does this pay off? Our survey was performed in order to provide at least some answers to these questions.

### Expert recommendation no. 3

Let us use a unique opportunity given by the European Commission via Horizon 2020 framework programme for the realisation of projects supporting personalised, predictive and preventive medicine, including innovation in medication processes. The Horizon 2020 work programme for years 2014–2015 declares that the Horizon 2020 societal challenge of ‘health, demographic change and wellbeing’ (SC1) for the years 2014 and 2015 includes 34 topics in ‘personalising health and care’ focus area call. The ‘personalising health and care’ call aims to create opportunities for real breakthrough research and radical innovation in response to these challenges by supporting the translation of findings into the clinic and other health and care settings to improve health outcomes, reduce health inequalities and promote active and healthy ageing. Usage of Horizon 2020 programme and possibly further programmes by EC for supporting EU health care development and innovation may remove the most frequently occurring obstacle, i.e. lack of financial sources.

The verification of personalised, predictive, preventive, safe and efficient medication potential cannot be done efficiently only on a national level. It needs an international cooperation, experience sharing, data exchange, support and understanding different attitudes to medication as well as to a hospital care. The European Commission offers, by means of its programmes, e.g. Horizon 2020, a unique opportunity to move ahead personalised, predictive and preventive medicine in terms of improvements to hospital medication safety and effectiveness. Should this article contribute to seeking or finding good arguments for innovation in medication process at both European-wide and national levels and/or to support making such things happen, this author's efforts made good sense.

## Endnote

^a^A predictive medication here means a kind of drug subscription predicting possible/relevant inter-reactions of chemical components of various drugs prescribed to a patient and administered simultaneously or within a short period in which they may inter-react. This term also applies to the prediction of the effects of these prescribed drugs on a patient based on the current/most recent patient's biochemical laboratory testing results.

## Competing interests

The author declares that he has no competing interests.

## Authors' information

ZV, MSC, MBA, has been working in top managerial positions in the consulting business for more than 15 years. Health and health care has been always one of the major industries he has been focusing on. He spent many years working for big international consulting companies. In the recent 3 years, he has been managing his own consulting company, ABC Works CZ s.r.o. (based in Prague, the Czech Republic) and working for Czech and Slovak clients prevailingly. ZV has managed a number of major projects for ministries of health, state health authorities/institutions, major health insurance companies, universities, big national and regional hospitals and private investors in health care. The ABC Works CZ s.r.o. is a member of the European Association for Predictive, Preventive & Personalised Medicine (please see http://www.epmanet.eu/). For more detailed information about the author's CV, please see http://www.epmanet.eu/images/stories/pdfs/veselik_cv.pdf; for more information about the ABC Works CZ s.r.o., please see http://www.abcworks.cz/ and/or http://en.abcworks.cz/. The below mentioned Ms. Michaela Veselikova, Bc. (MV), or Henrieta Pankova, MSC (HP), has been working for the ABC Works CZ. MV is the assistant to the Executive Director, and HP is the manager of the Company Grants & Incentives Department. Ms. Simona Plischke (SP) works as a business development manager in the HC Logic, s.r.o., a consulting company based in Ostrava, the Czech Republic (for more details, please see http://www.hc-logic.cz/).

## Supplementary Material

Additional file 1 This supplementary file contains tables on experts' opinions on the rate of medication errors, the rate of serious impacts caused by wrong medication and the economic impacts of medication errors. Click here for file
