# Supplementary material for: Personalised, predictive and preventive medication process in hospitals—still rather missing: professional opinion survey on medication safety in Czech hospitals (based on professional opinions of recognised Czech health care experts)
Source: EPMA J. 2014 May 1;5(1):7. doi: 10.1186/1878-5085-5-7 (PMC4021639; doi:10.1186/1878-5085-5-7)
Supplement: Additional file 1 — This supplementary file contains tables on experts' opinions on the rate of medication errors, the rate of serious impacts caused by wrong medication and the economic impacts of medication errors. [file 1878-5085-5-7-S1.pdf]

TABLES

All data in the below tables were collected from reputable Czech healthcare experts (cf. the chapter: Acknowledgements) in the period of September – November, 2013, and subsequently elaborated in the period of December, 2013 – January, 2014.

1. Tables providing experts opinions on the rate of medication errors

1.1 Table 1: **Answers of all respondents**

Full Wording of the Questions Asked	Total Number of Answers Received	Number of Answeres Received Sorted by Type		
		Definitely "YES"	Partially "YES"	"NO"
Is the Study, performed in the Städtisches Krankenhaus München-Harlaching (the "Study"), relevant to a real situation in Czech hospitals?	**29**	**26**	**3**	**0**
Is the Study methodology used for indentification of mistakes in prescription and administration of drugs trustfull, and logically correct?	**29**	**28**	**1**	**0**
Do you believe that the findings and conclusions of the Study apply to the situation in Czech hospitals?	**29**	**27**	**2**	**0**
		Lower Average	Upper Average	Mid Value of the Interval
What percentage interval of Adverse Drug Events of a medication of all inpatients in Czech hospitals do you consider trustfull and expectable in the Czech hospitals environment?	**28**	**4.37%**	**8.65%**	**6.51%**
The same interval after exclusion of one highest and one lowest values received	***26***	***3.51%***	***7.70%***	***5.61%***

1.2 Table 2: **Physicians' view**

Full Wording of the Questions Asked	Total Number of Answers Received	Number of Answeres Received Sorted by Type		
		Definitely "YES"	Partially "YES"	"NO"
Is the Study, performed in the Städtisches Krankenhaus München-Harlaching (the "Study"), relevant to a real situation in Czech hospitals?	**14**	**13**	**1**	**0**
Is the Study methodology used for indentification of mistakes in prescription and administration of drugs trustfull, and logically correct?	**14**	**13**	**1**	**0**
Do you believe that the findings and conclusions of the Study apply to the situation in Czech hospitals?	**14**	**14**	**0**	**0**
		Lower Average	Upper Average	Mid Value of the Interval
What percentage interval of Adverse Drug Events of a medication of all inpatients in Czech hospitals do you consider trustfull and expectable in the Czech hospitals environment?	**13**	**3.42%**	**6.62%**	**5.02%**
The same interval after exclusion of one highest and one lowest values received	***11***	***3.20%***	***6.74%***	***4.97%***

1.3 Table 3: **Nurses' view**

Full Wording of the Questions Asked	Total Number of Answers Received	Number of Answeres Received Sorted by Type		
		Definitely "YES"	Partially "YES"	"NO"
Is the Study, performed in the Städtisches Krankenhaus München-Harlaching (the "Study"), relevant to a real situation in Czech hospitals?	6	6	0	0
Is the Study methodology used for indentification of mistakes in prescription and administration of drugs trustfull, and logically correct?	6	6	0	0
Do you believe that the findings and conclusions of the Study apply to the situation in Czech hospitals?	6	6	0	0
		Lower Average	Upper Average	Mid Value of the Interval
What percentage interval of Adverse Drug Events of a medication of all inpatients in Czech hospitals do you consider trustfull and expectable in the Czech hospitals environment?	6	8.66%	16.49%	12.58%
The same interval after exclusion of one highest and one lowest values received	*4*	*5.24%*	*13.74%*	*9.49%*

1.4 Table 4: **Pharmacists' view**

Full Wording of the Questions Asked	Total Number of Answers Received	Number of Answeres Received Sorted by Type		
		Definitely "YES"	Partially "YES"	"NO"
Is the Study, performed in the Städtisches Krankenhaus München-Harlaching (the "Study"), relevant to a real situation in Czech hospitals?	5	3	2	0
Is the Study methodology used for indentification of mistakes in prescription and administration of drugs trustfull, and logically correct?	5	5	0	0
Do you believe that the findings and conclusions of the Study apply to the situation in Czech hospitals?	5	3	2	0
		Lower Average	Upper Average	Mid Value of the Interval
What percentage interval of Adverse Drug Events of a medication of all inpatients in Czech hospitals do you consider trustfull and expectable in the Czech hospitals environment?	5	2.18%	6.33%	4.26%
The same interval after exclusion of one highest and one lowest values received	*3*	*1.65%*	*3.83%*	*2.74%*

1.5 Table 5: **Non-medical managers' view**

Full Wording of the Questions Asked	Total Number of Answers Received	Number of Answeres Received Sorted by Type		
		Definitely "YES"	Partially "YES"	"NO"
Is the Study performed in the Städtisches Krankenhaus München-Harlaching (the "Study") relevant for a real situation within Czech hospitals?	4	4	0	0
Is the Study methodology used for indentification of mistakes in prescription and administration of drugs trustful and logically correct?	4	4	0	0
Do you believe that findings and conclusions of the Study apply to the situation within the Czech hospitals?	4	4	0	0
		Lower Average	Upper Average	Mid Value of the Interval
What percentage interval of Adverse Drug Events out of all medication of all inpatients in Czech hospitals would you consider trustful and expectable?	4	3.74%	6.24%	5.08%
The same interval after exclusion of one highest and one lowest values received		*3.98%*	*5.65%*	*4.82%*

2. TABLES PROVIDING EXPERT OPINIONS ON THE RATE OF SERIOUS IMPACTS CAUSED BY WRONG MEDICATION

2.1 Table 6: **Answers of all respondents**

Full Wording of the Questions Asked	Total Number of Answers Received	Number of Answeres Received Sorted by Type		
		Definitely "YES"	Partially "YES"	"NO"
Is the Study „MEDICATION ERRORS OBSERVED IN 36 HEALTH CARE FACILITIES“ (the „Study“) relevant toa real situation in Czech hospitals?	**29**	**23**	**4**	**1**
Is the Study methodology used for indentification of mistakes in medication and their consequences to patients health status trustfull and logically correct?	**29**	**26**	**2**	**0**
Do you believe that the Study findings or conclusions – i.e. medication mistakes and their influence on patiens health status – apply to the situation in Czech hospitals?	**29**	**23**	**5**	**0**
		Lower Average	Upper Average	Mid Value of the Interval
What percentage of interval of Adverse Drug Events having seriously negative influence on patients health status in the Czech hospitals would you consider trustfull and expectable in the Czech hospitals environment?	**28**	**5.01%**	**9.20%**	**7.11%**
The same interval after exclusion of one highest and one lowest values received	***26***	***5.00%***	***9.06%***	***7.03%***

2.2 Table 7: **Physicians' view**

Full Wording of the Questions Asked	Total Number of Answers Received	Number of Answeres Received Sorted by Type		
		Definitely "YES"	Partially "YES"	"NO"
Is the Study „MEDICATION ERRORS OBSERVED IN 36 HEALTH CARE FACILITIES“ (the „Study“) relevant toa real situation in Czech hospitals?	**14**	**13**	**1**	**0**
Is the Study methodology used for indentification of mistakes in medication and their consequences to patients health status trustfull and logically correct?	**14**	**12**	**2**	**0**
Do you believe that the Study findings or conclusions – i.e. medication mistakes and their influence on patiens health status – apply to the situation in Czech	**14**	**13**	**1**	**0**
		Lower Average	Upper Average	Mid Value of the Interval
What percentage of interval of Adverse Drug Events having seriously negative influence on patients health status in the Czech hospitals would you consider trustfull and expectable in the Czech hospitals environment?	**13**	**4.19%**	**8.55%**	**6.37%**
The same interval after exclusion of one highest and one lowest values received	***11***	***3.95%***	***7.38%***	***5.67%***

2.3 Table 8: **Nurses' view**

Full Wording of the Questions Asked	Total Number of Answers Received	Number of Answeres Received Sorted by Type		
		Definitely "YES"	Partially "YES"	"NO"
Is the Study „MEDICATION ERRORS OBSERVED IN 36 HEALTH CARE FACILITIES" (the „Study") relevant toa real situation in Czech hospitals?	**6**	**6**	**0**	**0**
Is the Study methodology used for indentification of mistakes in medication and their consequences to patients health status trustfull and logically correct?	**6**	**6**	**0**	**0**
Do you believe that the Study findings or conclusions – i.e. medication mistakes and their influence on patiens health status – apply to the situation in Czech hospitals?	**6**	**6**	**0**	**0**
		Lower Average	Upper Average	Mid Value of the Interval
What percentage of interval of Adverse Drug Events having seriously negative influence on patients health status in the Czech hospitals would you consider trustfull and expectable in the Czech hospitals environment?	**6**	**7.83%**	**14.33%**	**11.08%**
The same interval after exclusion of one highest and one lowest values received	***4***	***6.50%***	***14.00%***	***10.25%***

2.4 Table 9: **Pharmacists' view**

Full Wording of the Questions Asked	Total Number of Answers Received	Number of Answeres Received Sorted by Type		
		Definitely "YES"	Partially "YES"	"NO"
Is the Study „MEDICATION ERRORS OBSERVED IN 36 HEALTH CARE FACILITIES" (the „Study") relevant toa real situation in Czech hospitals?	**5**	**2**	**3**	**0**
Is the Study methodology used for indentification of mistakes in medication and their consequences to patients health status trustfull and logically correct?	**5**	**5**	**0**	**0**
Do you believe that the Study findings or conclusions – i.e. medication mistakes and their influence on patiens health status – apply to the situation in Czech hospitals?	**5**	**1**	**4**	**0**
		Lower Average	Upper Average	Mid Value of the Interval
What percentage of interval of Adverse Drug Events having seriously negative influence on patients health status in the Czech hospitals would you consider trustfull and expectable in the Czech hospitals environment?	**5**	**3.30%**	**5.00%**	**4.15%**
The same interval after exclusion of one highest and one lowest values received	***3***	***3.00%***	***6.67%***	***4.84%***

2.5 Table 10: **Non-medical managers' view**

Full Wording of the Questions Asked	Total Number of Answers Received	Number of Answeres Received Sorted by Type		
		Definitely "YES"	Partially "YES"	"NO"
Is the Study „MEDICATION ERRORS OBSERVED IN 36 HEALTH CARE FACILITIES" (the „Study") relevant toa real situation in Czech hospitals?	**4**	**4**	**0**	**0**
Is the Study methodology used for indentification of mistakes in medication and their consequences to patients health status trustfull and logically correct?	**4**	**4**	**0**	**0**
Do you believe that the Study findings or conclusions – i.e. medication mistakes and their influence on patiens health status – apply to the situation in Czech hospitals?	**4**	**4**	**0**	**0**
		**Lower Average**	**Upper Average**	**Mid Value of the Interval**
What percentage of interval of Adverse Drug Events having seriously negative influence on patients health status in the Czech hospitals would you consider trustfull and expectable in the Czech hospitals environment?	**4**	**5.58%**	**7.82%**	**6.70%**
The same interval after exclusion of one highest and one lowest values received		***7.22%***	***8.15%***	***7.69%***

3. TABLES PROVIDING EXPERT OPINIONS ON THE ECONOMIC IMPACTS OF MEDICATION ERRORS

3.1 Table 11: **Answers of all respondents**

Full Wording of the Questions Asked	Total Number of Answers Received	Number of Answeres Received Sorted by Type		
		Definitely "YES"	Partially "YES"	"NO"
Is the Study "COSTS OF ADVERSE DRUG EVENTS IN GERMAN HOSPITALS — A MICROCOSTING STUDY" (the „Study") relevant to a real situation in Czech hospitals?	**29**	**24**	**2**	**3**
Is the Study methodology used for indentification of extended average length of stay of hospital inpatients, due to serious Adverse Drug Events, trustful, and logically correct?	**29**	**24**	**1**	**3**
Do you believe that the Study findings, results, and conclusions, in terms of extended average length of stay of inpatients due to serious Adverse Drugh Events, apply to the situation in Czech hospitals?	**29**	**27**	**1**	**1**
		**Lower Average**	**Upper Average**	**Mid Value of the Interval**
What interval of extended average length of inpatients stay in hospital, due to serious Adverse Drug Events, would you consider trustful and expectable in the Czech hospitals environment?	**28**	**2.2**	**3.32**	**2.67**
The same interval after exclusion of one highest and one lowest values received	***25***	***2.6***	***3.11***	***2.59%***

3.2 Table 12: **Physicians’ view**

Full Wording of the Questions Asked	Total Number of Answers Received	Number of Answeres Received Sorted by Type		
		Definitely "YES"	Partially "YES"	"NO"
Is the Study "COSTS OF ADVERSE DRUG EVENTS IN GERMAN HOSPITALS — A MICROCOSTING STUDY" (the „Study“) relevant to a real situation in Czech hospitals?	14	10	2	2
Is the Study methodology used for indentification of extended average length of stay of hospital inpatients, due to serious Adverse Drug Events, trustful, and logically correct?	14	11	1	2
Do you believe that the Study findings, results, and conclusions, in terms of extended average length of stay of inpatients due to serious Adverse Drugh Events, apply to the situation in Czech hospitals?	14	12	1	1
		Lower Average	Upper Average	Mid Value of the Interval
What interval of extended average length of inpatients stay in hospital, due to serious Adverse Drug Events, would you consider trustful and expectable in the Czech hospitals environment?	13	1.92	2.92	2.42
The same interval after exclusion of one highest and one lowest values received	*10*	*2.00*	*2.91*	*2.46%*

3.3 Table 13: **Nurses’ view**

Full Wording of the Questions Asked	Total Number of Answers Received	Number of Answeres Received Sorted by Type		
		Definitely "YES"	Partially "YES"	"NO"
Is the Study "COSTS OF ADVERSE DRUG EVENTS IN GERMAN HOSPITALS — A MICROCOSTING STUDY" (the „Study“) relevant to a real situation in Czech hospitals?	6	5	0	1
Is the Study methodology used for indentification of extended average length of stay of hospital inpatients, due to serious Adverse Drug Events, trustful, and logically correct?	6	5	0	1
Do you believe that the Study findings, results, and conclusions, in terms of extended average length of stay of inpatients due to serious Adverse Drugh Events, apply to the situation in Czech hospitals?	6	6	0	0
		Lower Average	Upper Average	Mid Value of the Interval
What interval of extended average length of inpatients stay in hospital, due to serious Adverse Drug Events, would you consider trustful and expectable in the Czech hospitals environment?	6	2.13	4.63	3.38
The same interval after exclusion of one highest and one lowest values received	*4*	*2.20*	*3.73*	*2.97%*

3.4 Table 14: **Pharmacists' view**

Full Wording of the Questions Asked	Total Number of Answers Received	Number of Answeres Received Sorted by Type		
		Definitely "YES"	Partially "YES"	"NO"
Is the Study "COSTS OF ADVERSE DRUG EVENTS IN GERMAN HOSPITALS — A MICROCOSTING STUDY" (the „Study") relevant to a real situation in Czech hospitals?	5	5	0	0
Is the Study methodology used for indentification of extended average length of stay of hospital inpatients, due to serious Adverse Drug Events, trustful, and logically correct?	5	5	0	0
Do you believe that the Study findings, results, and conclusions, in terms of extended average length of stay of inpatients due to serious Adverse Drugh Events, apply to the situation in Czech hospitals?	5	5	0	0
		Lower Average	Upper Average	Mid Value of the Interval
What interval of extended average length of inpatients stay in hospital, due to serious Adverse Drug Events, would you consider trustful and expectable in the Czech hospitals environment?	5	2.16	3.38	2.77
The same interval after exclusion of one highest and one lowest values received	*3*	2.60	*3.00*	2.80

3.5 Table 15: Non-medical managers' view

Full Wording of the Questions Asked	Total Number of Answers Received	Number of Answeres Received Sorted by Type		
		Definitely "YES"	Partially "YES"	"NO"
Is the Study "COSTS OF ADVERSE DRUG EVENTS IN GERMAN HOSPITALS — A MICROCOSTING STUDY" (the „Study") relevant to a real situation in Czech hospitals?	4	4	0	0
Is the Study methodology used for indentification of extended average length of stay of hospital inpatients, due to serious Adverse Drug Events, trustful, and logically correct?	4	4	0	0
Do you believe that the Study findings, results, and conclusions, in terms of extended average length of stay of inpatients due to serious Adverse Drugh Events, apply to the situation in Czech hospitals?	4	4	0	0
		Lower Average	Upper Average	Mid Value of the Interval
What interval of extended average length of inpatients stay in hospital, due to serious Adverse Drug Events, would you consider trustful and expectable in the Czech hospitals environment?	4	1.98	2.98	2.48
The same interval after exclusion of one highest and one lowest values received		*1.95*	*2.45*	2.20
